# Lectures on the Principles of Surgery

**Published:** 1885-02

**Authors:** 


LECTURES ON THE PRINCIPLES OF SURGERY. *Delivered at Bellevue Hospital Medical College. By* W. H. VAN BUREN, M.D., LL.D. (Yalen), *formerly Professor of the Principles and Practice of Surgery in the Bellevue Hospital Medical College, etc., etc. Edited by* LEWIS A. STIMSON, M.D., *Professor of Physiology and Clinical Surgery in the Medical Department of the*

*University of the City of New York. Octavo, pp. vii, 588. New York:* D. Appelton & Co., 1884. *Chicago:* Jansen, McClurg & Co.

Dr. Van Buren, for a period of thirty-five years prior to his death, occupied a phenomenal position as a teacher of surgery. It was a matter of general regret, when he ceased from labor, that no imperishable form had been given to the charming lectures which, students of the Bellevue Hospital Medical College and Medical Department of the University of New York had heard with so much attention and profit Dr. Lewis A. Stimson has conferred a lasting benefit upon the profession by the conscientious discharge of his duty as editor of the late Professor Van Buren's lecture notes. As a tribute to the memory of a great American surgeon, and as an invaluable contribution to the literature of the subject, "Van Buren's Lectures on the Principles of Surgery" will find an honored place in every well-selected medical library.

